# Feasibility of Using a Multilingual Web Survey in Studying the Health of Ethnic Minority Youth

**DOI:** 10.2196/resprot.3655

**Published:** 2015-05-07

**Authors:** Jaana Maarit Kinnunen, Maili Malin, Susanna Ulrika Raisamo, Pirjo Liisa Lindfors, Lasse Antero Pere, Arja Hannele Rimpelä

**Affiliations:** ^1^School of Health SciencesUniversity of TampereTampereFinland; ^2^Child and Adolescent Mental Health UnitNational Institute for Health and WelfareHelsinkiFinland; ^3^Tobacco, Gambling and AddictionNational Institute for Health and WelfareHelsinkiFinland; ^4^Tampere Centre for Childhood, Youth and Family ResearchUniversity of TampereTampereFinland; ^5^Department of Adolescent PsychiatryTampere University HospitalTampereFinland

**Keywords:** feasibility, Web survey, ethnic minority, adolescents, response rate, representativeness, multilingual

## Abstract

**Background:**

Monolingual Web survey is a common tool for studying adolescent health. However, national languages may cause difficulties for some immigrant-origin youths, which lower their participation rate. In national surveys, the number of ethnic minority groups is often too small to assess their well-being.

**Objective:**

We studied the feasibility of a multilingual Web survey targeted at immigrant-origin youths by selection of response language, and compared participation in different language groups with a monolingual survey.

**Methods:**

The Adolescent Health and Lifestyle Survey (AHLS), Finland, with national languages (Finnish/Swedish) was modified into a multilingual Web survey targeted at a representative sample of 14- and 16-year olds (N=639) whose registry-based mother tongue was other than the national languages. The survey was conducted in 2010 (16-year olds) and 2011 (14-year olds). The response rate of the multilingual survey in 2011 is compared with the AHLS of 2011. We also describe the translation process and the e-form modification.

**Results:**

Of the respondents, 57.6% answered in Finnish, whereas the remaining 42.4% used their mother tongue (*P*=.002). A majority of youth speaking Somali, Middle Eastern, Albanian, and Southeast Asian languages chose Finnish. The overall response rate was 48.7% with some nonsignificant variation between the language groups. The response rate in the multilingual Web survey was higher (51.6%, 163/316) than the survey with national languages (46.5%, 40/86) in the same age group; however, the difference was not significant (*P*=.47). The adolescents who had lived in Finland for 5 years or less (58.0%, 102/176) had a higher response rate than those having lived in Finland for more than 5 years (45.1%, 209/463; *P*=.005). Respondents and nonrespondents did not differ according to place of birth (Finland/other) or residential area (capital city area/other). The difference in the response rates of girls and boys was nearly significant (*P*=.06). Girls of the Somali and Middle Eastern language groups were underrepresented among the respondents.

**Conclusions:**

A multilingual Web survey is a feasible method for gathering data from ethnic youth, although it does not necessarily yield a higher response rate than a monolingual survey. The respondents answered more often in the official language of the host country than their mother tongue. The varying response rates by time of residence, ethnicity, and gender pose challenges for developing tempting surveys for youth.

## Introduction

Population-based youth surveys to monitor young people’s health and health behaviors were established in the 1980s in many Western countries, such as the cross-national survey of Health Behaviour in School-Aged Children and the European School Survey Project on Alcohol and Drugs. In these national surveys, the immigrant-origin population often is too small to assess their well-being. Only recently, it has been realized that for developing health-promotion policies and programs, the general surveys do not necessarily give a reliable picture of the ethnically diversified youth population in Western countries [1-4]. There is a need for new tools to gather population data on health and health behaviors of ethnic minority youth.

It has been suggested that multilingualism in survey design is a basic precondition for improving participation and representativeness of different ethnic populations among adults [5,6]. Although the reliability and the quality of standardized interview data are shown to be better than those of postal or telephone surveys [7,8], it is not always a realistic method to be used nowadays due to its high cost and time-consuming nature. Many Western-born ethnic minority youths can respond to the surveys in their host country’s language. However, among those born outside their present host country, settlement age varies and their host country language skills might not be good enough to respond to a survey conducted in the host country’s language. Cultural values or anxiety about the use of the survey results might be further reasons for not taking part in surveys.

Few tailor-made surveys for ethnic minority youth have been conducted [1-3]. In a German cross-sectional study, 51% (1,479/2,900) of the sample aged 0-17 years having immigration background participated, compared with 68% (16,162/23,768) of the ethnic German population. Computer-assisted personal interview with a translated questionnaire and health examinations were performed on the participants in that study [1]. The participation rate in the Norwegian Youth, Culture and Competence study on ethnic minority minors’ health varied between substudies from 43.5% to 65% [2,4]. In the Danish Longitudinal Survey of Children study, 47.5% of the 12-year-old ethnic youths participated in standardized interpreted interviews and completed questionnaires [3]. In Finland, only one tailor-made interview study for immigrant-origin youth has been conducted so far, and the study included Somali- and Kurdish-speaking adolescents living in the capital city area [9].

In Finland, the largest immigrant groups come from Russia, Estonia, Somalia, Iraq, former Yugoslavia, and China [10]. Finland has undergone a rapid ethnic diversification during the last 20 years. However, even today, most immigrant-origin adults and their children are born abroad, and thus, belong to the first generation of immigrants [10]. In Finland, the age structure of people of foreign origin is different from those of Finnish origin: in 2011, the average age was younger among foreign-origin people (37.7 years) compared with Finnish-origin people (42.0 years), and the second-generation Finns with foreign origin are especially young (87% are under 18 years of age) [10]. In Finland, approximately half of the immigrants live in the Southern part of the country in the capital city area where approximately every 1 in 10 persons speaks a non-national language [11].

Little is known about the feasibility and use of multilingual Web surveys in studying health and health behaviors of ethnic minority youth in nationally representative samples. When adolescents are offered an option to use their mother tongue, we expect that their participation is not related to time lived in Finland, country of birth, or ethnicity and that mother tongue is a primary language when answering the survey. Further, we hypothesize that immigrant adolescents’ response rate is higher in multilingual surveys targeted specifically to them than in corresponding monolingual surveys targeted to all youths. In this study, we assess the feasibility of the multilingual Web survey targeted to ethnic minority adolescents in Finland using their officially registered mother tongue as a proxy for their ethnicity. First, we analyze the variation in response rates between ethnic groups according to gender, place of birth, time lived in Finland, residential area, and selection of survey language among 16- and 14-year-old adolescents. Second, we compare the response rate in this multilingual Web survey with the response rate in a corresponding survey carried out in the same year and same age group using only national languages.

## Methods

### Sample and Participants

The representative samples of 14- and 16-year olds whose registry-based mother tongue was other than one of the two official languages in Finland were drawn from the Population Register Centre, Finland, in 2010 (16-year olds) and 2011 (14-year olds). We combined these two data sets giving the total sample size of 800. Because of economic reasons and the small size of certain language groups, the original questionnaire was translated into the 12 most common foreign languages in Finland (Table 1), reducing the sample size from 800 to 639.

This study was built to correspond with the Adolescent Health and Lifestyle Survey (AHLS) database, a national monitoring system of adolescent health and health behaviors in Finland, in which self-administered questionnaires have only been available in the two official languages, Finnish and Swedish. The AHLS was established in 1977 and has been conducted once in every 2 years among nationally representative samples of 12-, 14-, 16-, and 18-year olds living in Finland. Samples have been obtained from the Population Register Center based on the particular dates of birth, so that all adolescents living in Finland and born on certain sample days in June, July, and August have been included in the surveys. From 2009 onward, respondents could have answered via either the Internet or a mailed questionnaire. The AHLS has been approved by the Ethical Committee of the Pirkanmaa Hospital District and by the Ethics Committee of the Tampere region. Details on the methodology of data collection using AHLS have been described elsewhere [12].

In the AHLS 2011, the number of 12-18-year-old ethnic minority youths was 383 (3.9% of the sample) and their response rate was 39.2% (150/383) compared with 47.1% (4,416/9,380) among those whose mother tongue was Finnish or Swedish (*P=*.002). For the comparison of the response rates, we used the data on 14-year olds in the AHLS 2011 of the same 12 language groups as the multilingual survey. The number of 14-year olds in the 12 language groups was 86. No data were available for 2010 as only the 16-year olds were surveyed.

We modified the AHLS into a multilingual Web survey, and the study participants were requested to answer in their preferred language. The self-administered questionnaire included 49 questions related to health behaviors (eg, smoking, use of alcohol, physical exercise, sleep, hobbies), health (eg, perceived health, stress symptoms), school, family, and religion. The vast majority of the questions were structured as *close-ended questions,* where respondents were given a list of answering options. Filling the questionnaire took approximately 30 minutes.

We classified adolescents into seven ethnic groups according to each person’s registered mother tongue. We used the language as a proxy of participants’ ethnicity. The groups were as follows: (1) Russian; (2) Estonian; (3) Somali; (4) Middle Eastern languages (Kurdish, Arabic, Persian, and Turkish); (5) Albanian; (6) Southeast Asian languages (Vietnamese, Chinese, and Thai); and (7) English. English-speaking youths could have come from different countries of origin, because in many past colonies of the Great Britain, English is still the official language. All participants in the Somali group and 9 of 10 in the Middle Eastern language group reported that they were Muslim.

### Translation Process

The AHLS gathers data on health and health behaviors using both online and paper questionnaires in Finnish. The questionnaire includes questions about sociodemographics, self-reported health, and health-related behaviors. Unfortunately, we were not able to follow the key recommendations and guidelines regarding a valid and rigorous translation process (ie, backtranslation) fully in our study [13,14]. Translations were based on the English version of the questionnaire, which was first given to public health university students who were native speakers of the selected languages. They were informed about the objectives and target age groups of the study. The translated questionnaires were then revised by professional reviewers in the Finnish official translation center. Kurdish, Albanian, and Thai questionnaires were translated only in the translation center because there were no native speakers of these languages among the students. Professional translators checked that the texts were linguistically correct and understandable. Both professional translators and native-speaker students were consulted on whether or not different dialects of Persian, Kurdish, Chinese, or Arabian needed to be considered in the translation process. Because they both answered “No,” only some minor revisions were made. Finally, professional translators translated the invitation letters and reminder notifications.

### E-form Modification

We used a common Web-based e-form platform, which is designed for conducting Web-based surveys. The system allows users to choose the preferred language when answering the questionnaire. From the e-form, the collected information was easily transferrable into spreadsheets and statistics software. Because of software limitations, the original questionnaire was slightly modified concerning the layout of questions and how the answers were to be chosen (ie, drop-down scale). Owing to the technical difficulties in using non-Latin alphabets in the e-form platform, translated paper questionnaires were also created for Russian, Kurdish, Arabic, Persian, Vietnamese, Thai, and Chinese language groups. In 2010, these paper questionnaires were sent along with the first reminder so that the respondents could read the questionnaire in their mother tongue when they had problems typing with the non-Latin alphabet in the e-form. The respondents were informed to return the paper questionnaire by mail in prestamped envelopes. This possibility was not available in 2011 because very few respondents used paper questionnaires in 2010.

### Data Collection and Processing

We mailed an invitation letter to the sample of 16-year-old youths in June-August 2010 and to the 14-year-old youths in March-June 2011. Participants received a letter both in their mother tongue and in Finnish. The introductory letter pointed out the confidentiality and the voluntary nature of the survey participation. Along with the invitation letter, a website address and a unique user ID and password were distributed. The minimum requirement for participation was access to the Internet. Nonrespondents were reminded two times via mailed letters. In 2010, the second reminder included a questionnaire asking for a reason for the refusal.

Results were exported from the e-form software to an Excel spreadsheet (Microsoft, Richmond, VA, USA). All personal identifiers were removed from the research file. After the Excel modifications, the data were analyzed using IBM SPSS Statistics, version 20 (IBM Corp, Armonk, NY, USA). All variables used in this study were categorical. The following categories were used in the analyses: gender (boy vs girl), place of birth (abroad vs Finland), time lived in Finland (≤5 years vs >5 years), and residential area (capital city area vs other). Statistical differences between groups were tested using Fisher exact test (two tailed).

## Results

The overall response rate of the multilingual Web survey (N=639) was 48.7% (311/639). There was some variation in the response rates between the language groups but the differences were not significant (*P*=.58; Table 1). A total of 15 adolescents who actively refused to participate in the study gave reasons for doing so. The most common reasons were “I don’t want to”/“I don’t feel like answering,” “Lack of time,” and “I am Finnish”/“I don’t feel like belonging to the target group.”

**Table 1:** Sample size, number of respondents, response rate, and the distribution of language groups among sample and respondents.

Language group	Sample size N	Number of respondents	Response rate (%)	Sample (%)	Respondents (%)
Russian	175	84	48.0	27.4	27.0
Estonian	87	49	56.3	13.6	15.8
Somali	86	37	43.0	13.5	11.9
Middle Eastern languages^a^	145	65	44.8	22.7	20.9
Albanian	46	24	52.2	7.2	7.7
Southeast Asian languages^b^	73	38	52.1	11.4	12.2
English	27	14	51.9	4.2	4.5
Total	639	311	48.7	100.0	100.0

^a^Kurdish, Arabic, Persian, and Turkish.

^b^Vietnamese, Chinese, and Thai.

The response rate of the 14-year olds in the 2011 multilingual survey was compared with the corresponding age group in the monolingual AHLS carried out in the same year. The response rate in the multilingual survey was higher (51.6%, 163/316) than in the monolingual survey (46.5%, 40/86) but the difference was not statistically significant (*P*=.47).

With regard to gender, girls participated more actively in the survey than boys: the response rate for girls was 52.5% (167/318) and that for boys was 44.9% (144/321). The difference was nearly statistically significant (*P*=.06). When only one language group was studied at a time, the response rate of girls was significantly higher only among the Southeast Asian language group (66.7%, 26/39 vs 35.3%, 12/34; *P*=.01) and English-speaking youth (80.0%, 8/10 vs 35.3%, 6/17; *P*=.046).

The response rate for those living in the capital city area was lower (45.1%, 119/264) than for those living in other areas of Finland (51.2%, 192/375) but the difference was not statistically significant (**P*=.*15). When only one language group was studied at a time, only Russian-speaking youth had a significantly lower response rate among those living in the capital city area (36.7%, 22/60) than in the other areas (53.9%, 62/115; *P*=.04).

The response rate for adolescents born abroad was higher (50.1%, 212/423) compared with the response rate for those born in Finland (45.8%, 99/216) but the difference was not statistically significant (*P*=.32). When only one language group was studied at a time, only the Middle Eastern language group had a significantly higher response rate among those born abroad (50.0%, 54/108 vs 29.7%, 11/37; *P*=.04).

The response rate for those settled in Finland during the previous 5 years was 58.0% (102/176), whereas it was 45.1% (209/463) for those who had lived in Finland for more than 5 years. The difference was statistically significant (*P*=.005). When only one language group was studied at a time, only the Middle Eastern language group had a higher response rate among those having lived in Finland for 5 years or less (74.2%, 23/31 vs 36.8%, 42/114; *P*=.001).

**Figure 1:**
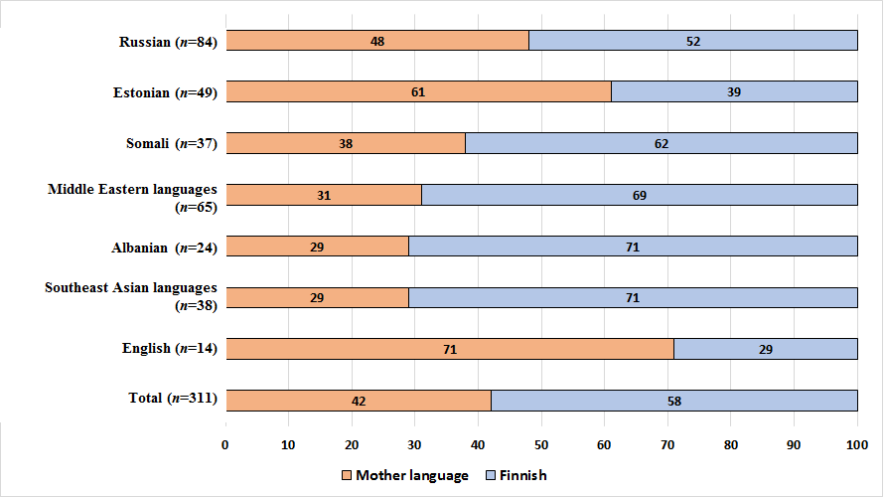
Response language (%) in multilingual Web survey by language group.

As many as 57.6% (179/311) of the youths responded in Finnish and 42.4% (132/311) responded in their registered mother tongue (Figure 1). There was variation in the selection of the survey language according to ethnicity (*P*=.002). Most English- and Estonian-speaking adolescents answered in their mother tongue, whereas a majority of the youth speaking Somali, Middle Eastern, Albanian, and Southeast Asian languages chose Finnish.

Tables 2 and 3 present the language group distribution between respondents and nonrespondents, that is, whether some groups are overrepresented or underrepresented among the respondents when compared with the nonrespondents. The respondents and nonrespondents are compared by gender, residential area, country of birth, and time lived in Finland.

Estonian, Southeast Asian languages, and English-speaking girls were overrepresented among the respondents, whereas girls speaking Somali and Middle Eastern languages were underrepresented (Table 2). The difference between the respondents and nonrespondents was statistically significant (*P*=.01). Among boys, difference in the distribution of language groups between the respondents and nonrespondents was not statistically significant (*P*=.69). Differences in distribution of language groups between the respondents and nonrespondents also were not statistically significant among those living in the capital city area (*P*=.36), or elsewhere in Finland (*P*=.65), among those born abroad (*P*=.82) or born in Finland (*P*=.10), nor among those who had settled in Finland during the last 5 years (*P*=.43) or before (*P*=.12; Tables 2 and 3).

**Table 2:** Distribution of language groups among respondents and nonrespondents according to gender and residential area, % (n), and *P* value for differences between respondents and nonrespondents.

Language group	Gender				Residential area			
	Boys (n=321)		Girls (n=318)		Capital city area (n=264)		Other (n=375)	
	Responded		Responded		Responded		Responded	
	Yes % (n)	No % (n)	Yes % (n)	No % (n)	Yes % (n)	No % (n)	Yes % (n)	No % (n)
Russian	25.0 (36)	27.7 (49)	28.7 (48)	27.8 (42)	18.5 (22)	26.2 (38)	32.3 (62)	29.0 (53)
Estonian	14.6 (21)	13.0 (23)	16.8 (28)	9.9 (15)	18.5 (22)	14.5 (21)	14.1 (27)	9.3 (17)
Somali	15.3 (22)	13.0 (23)	9.0 (15)	17.2 (26)	18.5 (22)	24.8 (36)	7.8 (15)	7.1 (13)
Middle Eastern languages^a^	25.7 (37)	23.7 (42)	16.8 (28)	25.2 (38)	16.0 (19)	15.9 (23)	24.0 (46)	31.1 (57)
Albanian	6.9 (10)	4.0 (7)	8.4 (14)	9.9 (15)	10.9 (13)	8.3 (12)	5.7 (11)	5.5 (10)
Southeast Asian languages^b^	8.3 (12)	12.4 (22)	15.6 (26)	8.6 (13)	12.6 (15)	7.6 (11)	12.0 (23)	13.1 (24)
English	4.2 (6)	6.2 (11)	4.8 (8)	1.3 (2)	5.0 (6)	2.8 (4)	4.2 (8)	4.9 (9)
Total	100 (144)	100 (177)	100 (167)	100 (151)	100 (119)	100 (145)	100 (192)	100 (183)
*P* value	.69		.01		.36		.65	

^a^Kurdish, Arabic, Persian, and Turkish.

^b^Vietnamese, Chinese, and Thai.

**Table 3:** Distribution of language groups among respondents and nonrespondents according to place of birth and time lived in Finland, % (n), and *P* value for differences between respondents and nonrespondents.

Language group	Place of birth				Time lived in Finland			
	Abroad (n*=*423)		Finland (n*=*216)		≤5 years (n*=*176)		>5 years (n*=*463)	
	Responded		Responded		Responded		Responded	
	Yes % (n)	No % (n)	Yes % (n)	No % (n)	Yes % (n)	No % (n)	Yes % (n)	No % (n)
Russian	31.6 (67)	35.1 (74)	17.2 (17)	14.5 (17)	30.4 (31)	35.1 (26)	25.4 (53)	25.6 (65)
Estonian	16.0 (34)	11.4 (24)	15.2 (15)	12.0 (14)	18.6 (19)	20.3 (15)	14.4 (30)	9.1 (23)
Somali	9.9 (21)	9.0 (19)	16.2 (16)	25.6 (30)	14.7 (15)	16.2 (12)	10.5 (22)	14.6 (37)
Middle Eastern languages^a^	25.5 (54)	25.6 (54)	11.1 (11)	22.2 (26)	22.5 (23)	10.8 (8)	20.1 (42)	28.3 (72)
Albanian	4.7 (10)	5.2 (11)	14.1 (14)	9.4 (11)	0.0 (0)	1.4 (1)	11.5 (24)	8.3 (21)
Southeast Asian languages^b^	9.9 (21)	10.0 (21)	17.2 (17)	12.0 (14)	9.8 (10)	9.5 (7)	13.4 (28)	11.0 (28)
English	2.4 (5)	3.8 (8)	9.1 (9)	4.3 (5)	3.9 (4)	6.8 (5)	4.8 (10)	3.1 (8)
Total	100 (212)	100 (211)	100 (99)	100 (117)	100 (102)	100 (74)	100 (209)	100 (254)
*P* value	.82		.10		.43		.12	

^a^Kurdish, Arabic, Persian, and Turkish.

^b^Vietnamese, Chinese, and Thai.

## Discussion

Nearly half of the respondents used their own mother tongue in filling out the questionnaire. The overall response rate was 48.7% with some nonsignificant variation between the language groups. The overall response rate did not vary statistically significantly by place of residence or country of birth but youth who had lived in Finland for 5 years or less participated more actively than those settled in Finland over 5 years ago; the higher response rate of girls was statistically nearly significant (*P*=.06) when compared with boys. Some significant differences were observed when studying each language group separately. Among the girls, youths speaking Somali and Middle Eastern languages were underrepresented among the participants.

The multilingual survey made a difference among ethnic minority youth, because as many as 42.4% of the respondents filled in the questionnaire in their mother tongue. There was variation among respondents in terms of their ethnic background. Most Estonian- and English-speaking youths and nearly half of the Russian respondents answered in their mother language, whereas most youths in other language groups selected the Finnish language questionnaire. One explanation for filling the survey in Finnish could be the technical problems with the non-Latin alphabets of Persian, Kurdish, Chinese, Thai, and Vietnamese languages in Western-based software [15]. It could be that these youths were reading the paper questionnaire in their mother tongue while filling out the Web survey in Finnish.

Another explanation for filling out the survey in Finnish could be that some of these youth’s language skills in Finnish are better than in their mother tongue. Most first- and second-generation ethnic minority adolescents can speak their mother language well but some of them may not be able to read and write well enough to answer a survey [16]. At home, adolescents may communicate in their mother tongue with their parents and siblings, whereas the selection of language at school with peers may depend on the context.

Some adolescents could have identified themselves more as Finnish-speaking youths or Finnish youths in this study context, rather than according to their ethnic family background, which was indicated in the invitation letter as a reason for sampling in the study. This might explain why they answered in Finnish and not in their mother language and also explain the higher response rate among those who had settled in Finland during the last 5 years compared with those who had lived in Finland for over 5 years.

Women usually participate more actively than men in surveys [17]. The same was observed in our survey although the difference was only nearly statistically significant (*P*=.06). Girls having their roots in Muslim countries were underrepresented among the respondents. Religion was asked in the questionnaire, and all Somalis and 9/10 in the Middle Eastern language group were Muslim. Somalis are the largest African-origin and Muslim-immigrant group in Finland [10], and health-related studies concerning the Finnish Somali population had been conducted recently, which might have negatively affected their willingness to participate in this survey. In addition, increased hate talk against Somalis and other African-origin immigrant groups on the Internet may have decreased their willingness to participate [18]. Further, culturally determined and gendered survey behavior could have affected Muslim girls’ unwillingness to participate. In a Finnish national school survey, the number of participating girls with Somali and Iraqi background was remarkably lower than that of boys even though their numbers in the school population are approximately the same [19]. The actual response rates could not be calculated in that survey.

Our study was the first population-based study for ethnic minority adolescents in Finland that used a multilingual Web survey. However, the response rate of ethnic minority youth was rather low. This is a common problem in surveys today regardless of the ethnicity of the respondents and it seems that, like our survey, non-school-based surveys also achieve approximately only 50% response rates among ethnic minority youths [1,3,4].

We hypothesized that immigrant adolescents’ response rate would be higher in a multilingual survey than in a monolingual survey. We could compare only 14-year olds whose response rate was higher (51.6%) compared with participants in the same age and language groups in the monolingual survey (46.5%); however, the difference was not statistically significant (*P*=.47). Larger sample numbers are needed to diminish the possibility of chance variation.

For the sampling of ethnic minority youth, we used registered mother language information from the national population registry. The mother language is reported by parents when the child is born or when moving to Finland. Registry-based mother language as a proxy for ethnicity is the least problematic of the proxies normally used; nationality excludes immigrants who have Finnish nationality, and country of birth includes Finns who were born abroad but moved back to Finland. However, it does not necessarily correspond to ethnicity defined by an adolescent himself/herself. Self-defined ethnicity could not be used as a basis for sampling as such a kind of information does not exist in the registry, and this information was not asked in the questionnaire either. Lack of a cross-cultural validity of the translated questionnaires is a significant limitation in studies like this. We cannot exclude validity problems in our study. By contrast, in this study, we used only registry-based data on gender, place of residence, country of birth, and time lived in Finland as well as the language of the questionnaire for analysis. A further limitation of our study was that for economic reasons we could not translate questionnaires to all minority languages. This may have had effects, for example, on the overall response rates.

Internet-based surveys are increasingly used and considered to be a supplement or an alternative to traditional postal surveys. Our results show that a Web survey can be considered a relevant and valid survey method for studying ethnic youth. The advantages of the Web survey are that it is cheaper, eliminates mailing procedure, is faster in transmission of data, and is environmentally friendlier [15,20,21]. In terms of reliability, electronic surveys may reduce social desirability bias and eliminate interviewer effect [22]. In the United States, 13-17-year-old students evaluated an Internet-based health questionnaire positively and it resulted in equal scores of health status or health behavior compared with the paper-and-pencil model [23]. Especially among youth, the Internet is the most promising tool as a data-collection vehicle, because they have been the early adopters of the rapidly growing Internet in most countries [22,24-26]. In Finland, nearly every adolescent has access to the Internet and most have a mobile phone enabling direct access to the Internet [27]. Adolescents have grown up with this technology and it appears to be an integral part of their daily lives. Beside many positive aspects, Internet-based health surveys also pose challenges [22,28] in an age when survey participation rates have declined significantly everywhere. All things considered, a multilingual Web survey with specific ethnicity-related questions can be recommended for collecting data on health and well-being of ethnic minority youth. Such a survey can be linked with national youth surveys by oversampling of immigrant-origin youth and creating optional Internet-based questionnaires in all relevant languages.

To conclude, multilingual Web survey is a feasible method for gathering data from ethnic youth, although several questions need to be scrutinized when developing tempting surveys for youth. First, the multilingual survey is not a self-evident guarantee for a higher response rate than in monolingual national surveys. Response rate seems to vary according to ethnic background, gender and the time of residence in the host country. The longer the youths have lived in the host country, the less likely they are to answer the multilingual survey, and thus, the self-defined ethnicity might play a role in terms of participation. Second, the youths do not automatically respond in their mother tongue, but may prefer to answer in the official language of the host country.

## Abbreviations

AHLS: Adolescent Health and Lifestyle Survey
